# Supplementary material for: Using insurance data to quantify the multidimensional impacts of warming temperatures on yield risk
Source: Nat Commun. 2020 Sep 11;11:4542. doi: 10.1038/s41467-020-17707-2 (PMC7486405; doi:10.1038/s41467-020-17707-2)
Supplement: Supplementary file 1 — Supplementary Information [file 41467_2020_17707_MOESM1_ESM.pdf]

Supplementary Information for

Using Insurance Data to Quantify the Multidimensional Impacts of Warming Temperatures on Yield Risk

Perry et al.

May 31, 2020

Supplementary Figures

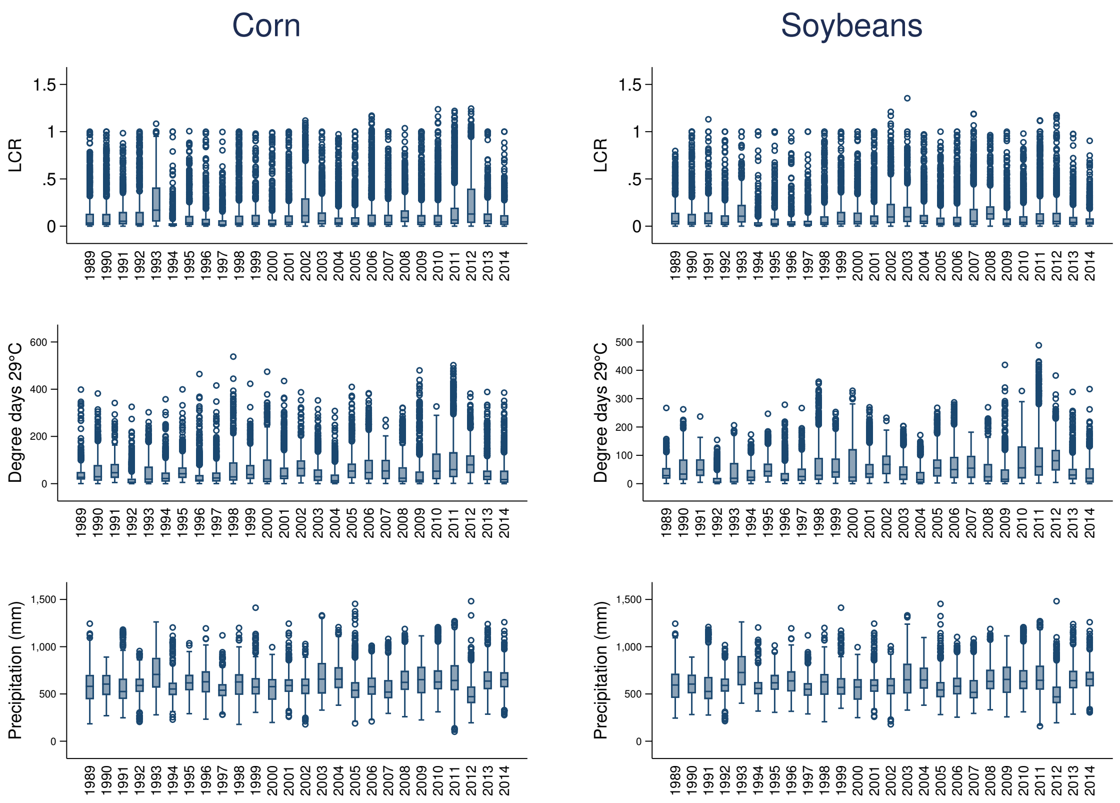

]

Supplementary Figure 1: Distribution of county-level average loss-cost ratios (LCR), degree days above 29°C, and precipitation, 1989-2014 (N=30,261 for corn and N=29,014 for soybeans). The minima and maxima of the boxes represent the 25% and 75% percentiles (q_1 and q_3), and the center of the boxes represents the median (q_2). The whiskers extend the boxes to the 1.5 inter-quartile range (IQR). The lower and upper bounds of the whiskers are $q_1 - 1.5$IQR and $q_2 + 1.5$IQR. Individual points are outside 1.5 IQR.

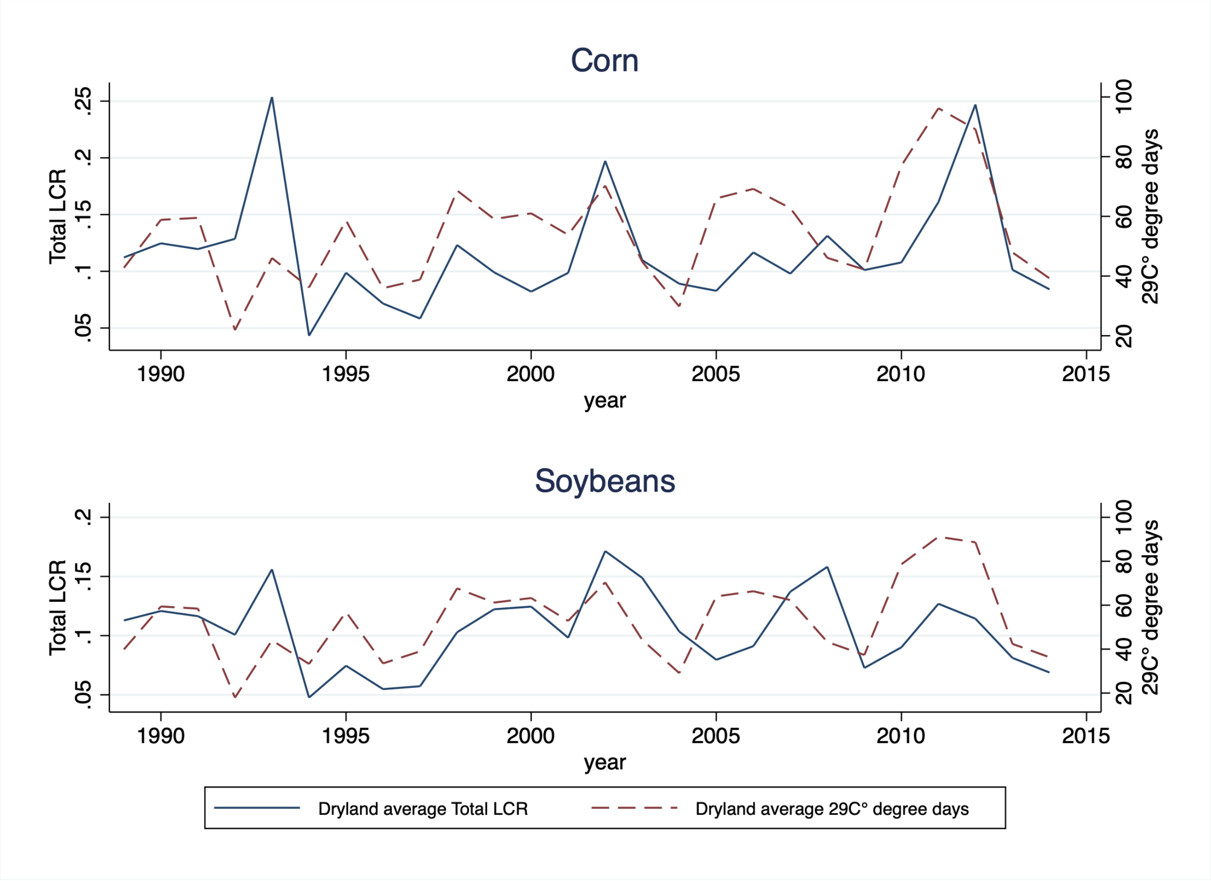

Supplementary Figure 2: Average total LCR across U.S. dryland counties, 1989-2014 (N=30,261 for cor, and N=29,014 for soybeans). Sample averages are calculated each year from the corn and soybean samples.

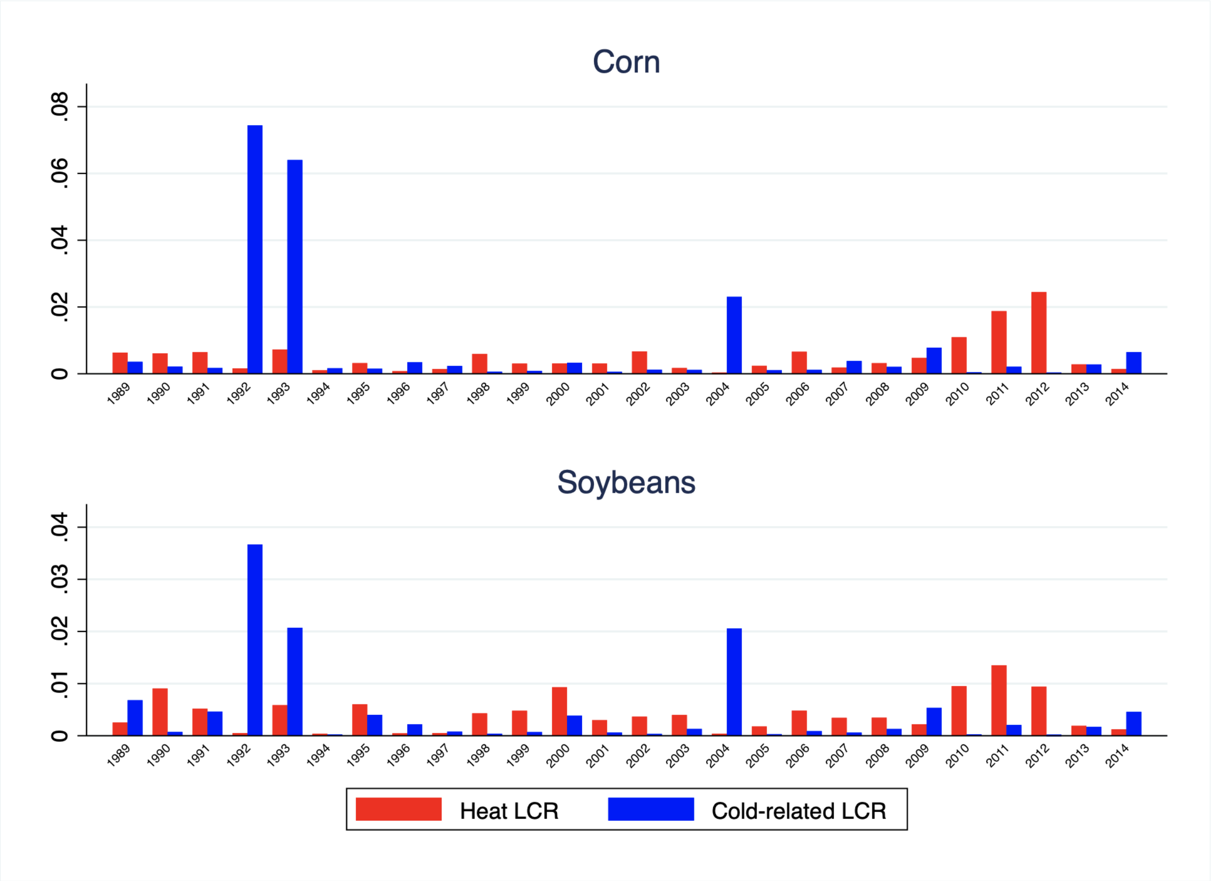

Supplementary Figure 3: Average LCRs due to heat (excluding drought) or cold across U.S. dryland counties, 1989-2014 (N=30,261 for corn and N=29,014 for soybeans). Sample averages are calculated each year from the corn and soybean samples.

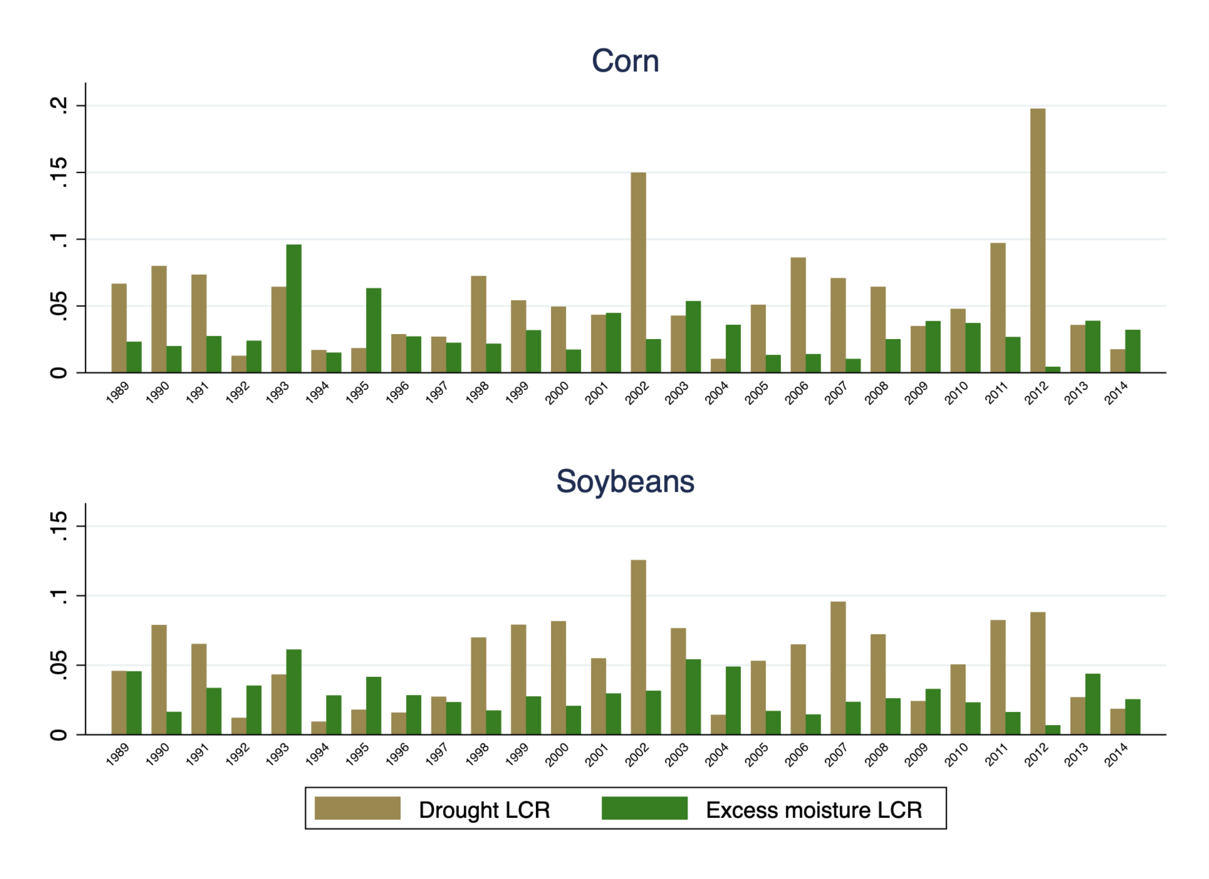

Supplementary Figure 4: Average LCRs due to drought or excess moisture across U.S. dryland counties, 1989-2014 (N=30,261 for corn and N=29,014 for soybeans). Sample averages are calculated each year from the corn and soybean samples.

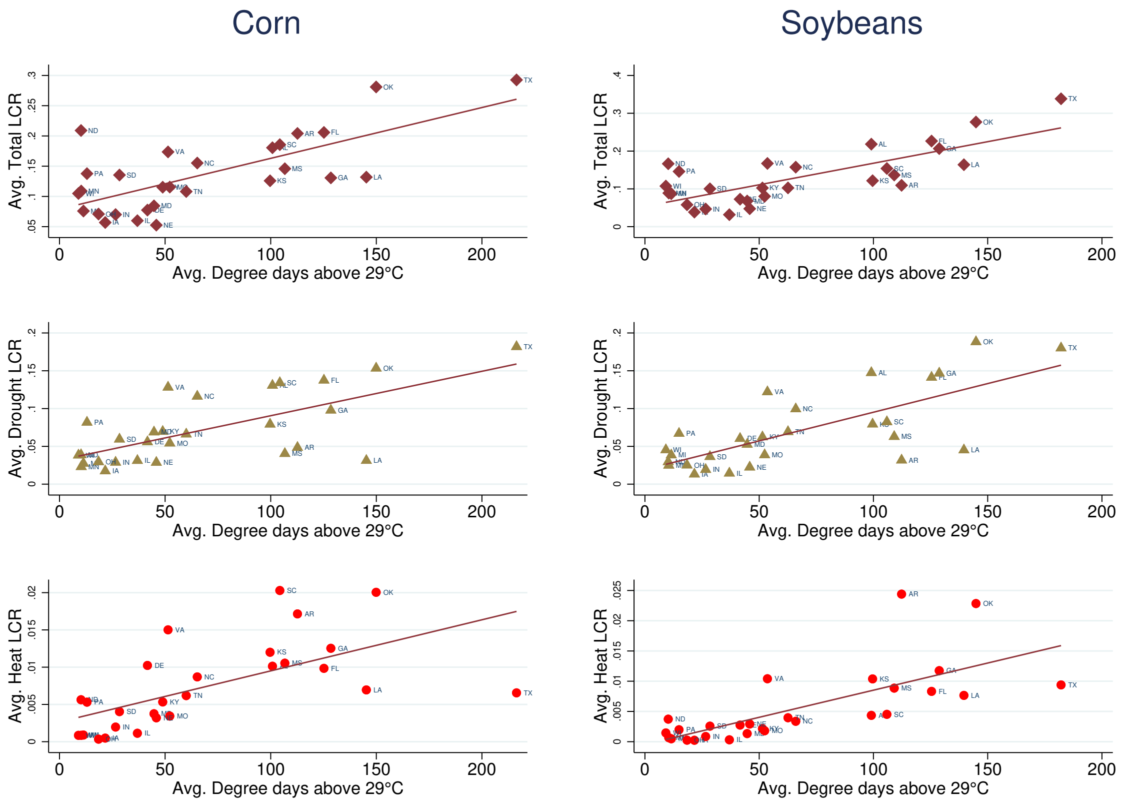

Supplementary Figure 5: Mean loss-cost ratio (LCR) and average number of degree days above 29°C by U.S. state, 1989 - 2014 (N=30,261 for corn and N=29,014 for soybeans). The regression line represents the best linear fit (N=28 states).

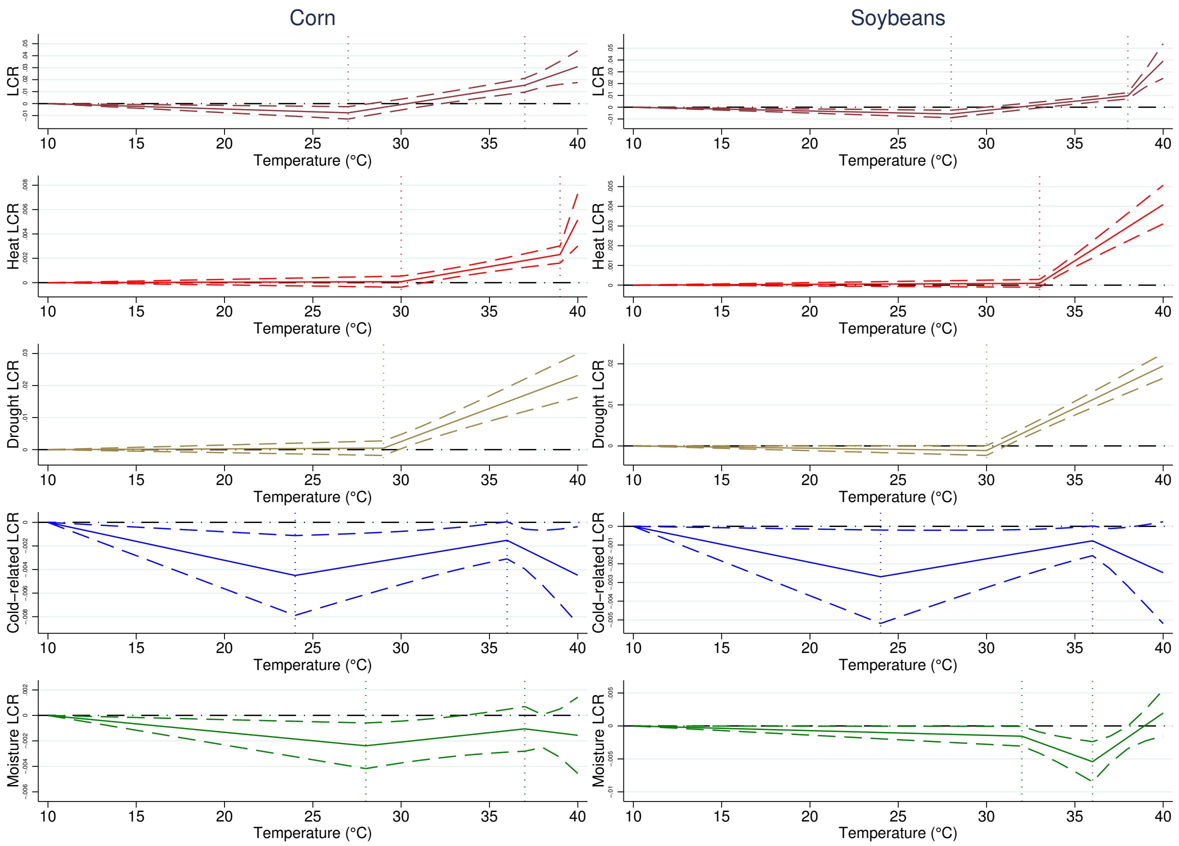

Supplementary Figure 6: Marginal effects of temperature on the aggregate and cause-specific LCRs (N=30,261 for corn and N=29,014 for soybeans). Dashed lines represent 95% confidence intervals and standard errors are clustered by year. For the drought LCRs and soybean heat LCR, the second cutoffs exceed 40 °C and the marginal effects in the last temperature intervals are too large to be reported.

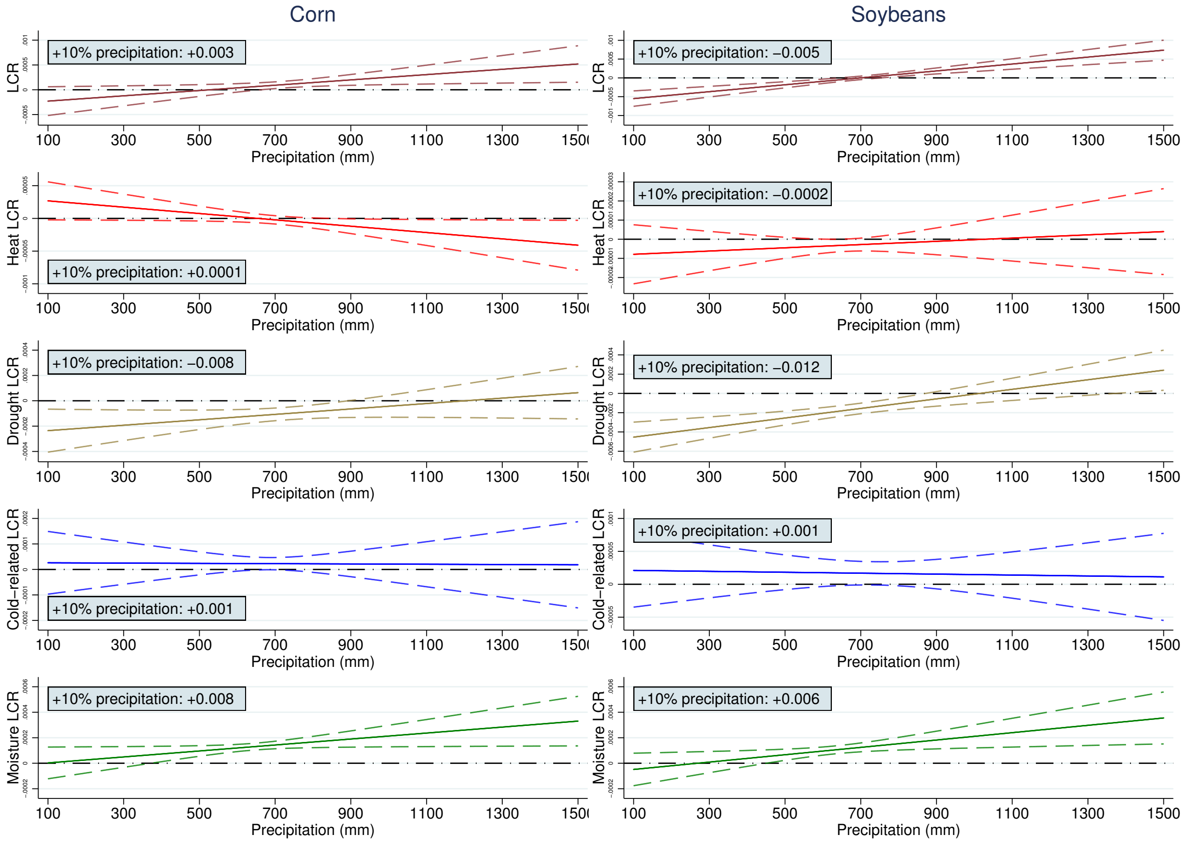

Supplementary Figure 7: Marginal effects of precipitation on the aggregate and cause-specific LCRs (N=30,261 for corn and N=29,014 for soybeans). Dashed lines represent 95% confidence intervals. Standard errors are clustered by year. The impacts of a 10% increase in precipitation on the aggregate and cause-specific LCRs are reported in the blue boxes. Note that the marginal effects are not statistically significant for the heat and cold-related LCRs.

Supplementary Tables

Supplementary Table 1: Goodness-of-fit, Temperature Cutoffs, and Warming Impacts (N= 30,261 for Corn, and N=29,014 for Soybeans)

	LCR	Heat	Cold-related	Drought	Excess Moisture
			R^2		
Corn	0.496	0.278	0.308	0.516	0.417
Soybeans	0.555	0.283	0.247	0.571	0.353
			Cutoffs		
Corn	(27, 37)	(30, 39)	(24, 36)	(29, 43)	(28, 37)
Soybeans	(28, 38)	(33, 41)	(24, 36)	(30, 41)	(32, 36)
			Warming Impacts		
Corn	0.0381**	0.00587***	-0.0167**	0.0570***	-0.0129**
	(0.0167)	(0.00132)	(0.00658)	(0.0104)	(0.00566)
Soybeans	0.0115	0.00458***	-0.00928**	0.0331***	-0.0171***
	(0.00962)	(0.000708)	(0.00416)	(0.00457)	(0.00560)

Note: Cold-related losses are the sum of freeze, frost, and cold wet weather. Standard errors, in parentheses, are clustered by year (*** p<0.01, ** p<0.05, * p<0.1, where the p-values are from a two-sided t-test).

Supplementary Table 2: Robustness Checks - Alternative Measures of Temperature Variables (N= 30,261 for Corn, and N=29,014 for Soybeans)

	LCR	Heat	Cold-related	Drought	Excess Moisture
			Warming Impacts		
Including Colder Temperatures					
Corn	0.0413**	0.00566***	-0.0166**	0.0595***	-0.0119**
	(0.0168)	(0.00137)	(0.00660)	(0.0101)	(0.00545)
Soybeans	0.00847	0.00420***	-0.00928**	0.0273***	-0.0143**
	(0.0108)	(0.000701)	(0.00446)	(0.00523)	(0.00593)
Alternative Growing Season (Mar. – Oct.)					
Corn	0.0497**	0.00584***	-0.0150**	0.0660***	-0.0121**
	(0.0193)	(0.00160)	(0.00717)	(0.0134)	(0.00587)
Soybeans	0.0262**	0.00455***	-0.00926**	0.0450***	-0.0151**
	(0.0105)	(0.000755)	(0.00468)	(0.00650)	(0.00595)
Alternative Growing Season (Mar. – Oct.) & Including Colder Temperatures					
Corn	0.0484**	0.00538***	-0.0179**	0.0683***	-0.0118*
	(0.0189)	(0.00173)	(0.00821)	(0.0127)	(0.00627)
Soybeans	0.0253**	0.00452***	-0.0109*	0.0400***	-0.0105*
	(0.0100)	(0.000711)	(0.00569)	(0.00634)	(0.00619)

Note: Cold-related losses are the sum of freeze, frost, and cold wet weather. Standard errors, in parentheses, are clustered by year (*** p<0.01, ** p<0.05, * p<0.1, where the p-values are from a two-sided t-test).

Supplementary Table 3: Robustness Checks - Average LCR Warming Impacts for Different Insurance Policies

	Corn					
Causes	All Policies		Yield Policies		Revenue Policies	
	Observed LCR	Warming Impact	Observed LCR	Warming Impact	Observed LCR	Warming Impact
Total LCR	0.119	0.0381**	0.100	0.0263*	0.142	0.0633***
		(0.0167)		(0.0140)		(0.0207)
Total LCR Excluding Price Losses	0.116	0.0366**	-	-	0.136	0.0609***
		(0.0162)		-		(0.0198)
Drought	0.0619	0.0570***	0.0530	0.0488***	0.0758	0.0681***
		(0.0104)		(0.00814)		(0.0144)
Excess Moisture	0.0298	-0.0129**	0.0257	-0.0135**	0.0351	-0.00757*
		(0.00566)		(0.00610)		(0.00398)
Heat	0.00564	0.00587***	0.00444	0.00484***	0.00713	0.00631***
		(0.00132)		(0.00118)		(0.00197)
Cold-related	0.00653	-0.0167**	0.00641	-0.0160**	0.00422	-0.0115
		(0.00658)		(0.00662)		(0.00728)
No. of Counties	1,733		1,697		1,697	

	Soybeans					
Causes	All Policies		Yield Policies		Revenue Policies	
	Observed LCR	Warming Impact	Observed LCR	Warming Impact	Observed LCR	Warming Impact
Total LCR	0.107	0.0115	0.0919	0.00892	0.124	0.0134
		(0.00962)		(0.00757)		(0.0146)
Total LCR Excluding Price Losses	0.104	0.0122	-	-	0.120	0.0143
		(0.00936)		-		(0.0141)
Drought	0.0562	0.0331***	0.0480	0.0282***	0.0689	0.0344***
		(0.00457)		(0.00450)		(0.00899)
Excess Moisture	0.0291	-0.0171***	0.0265	-0.0157***	0.0319	-0.0192***
		(0.00560)		(0.00540)		(0.00597)
Heat	0.00437	0.00458***	0.00338	0.00366***	0.00554	0.00500***
		(0.000708)		(0.000669)		(0.000936)
Cold-related	0.00396	-0.00928**	0.00349	- 0.00767***	0.00308	-0.00888
		(0.00416)		(0.00280)		(0.00608)
No. of Counties	1,632		1,600		1,614	

Note: Cold-related losses are the sum of losses from freeze, frost, and cold wet weather. For corn, there are 36 counties that only participated in yield policies and another 36 counties that only participated in revenue policies. For soybeans, there are 18 counties that only participated in yield policies and another 32 counties that only participated in revenue policies. Standard errors, in parentheses, are clustered by year (*** p<0.01, ** p<0.05, * p<0.1, where the p-values are from a two-sided t-test).

Supplementary Table 4: Robustness Checks - Out-of-sample Performances and Average LCR Warming Impacts with Additional Control Variables

Additional Control	Corn		Soybeans	
	Out-of-sample RMSE	Warming Impact	Out-of-sample RMSE	Warming Impact
No Additional Control	0.190	0.0381**	0.491	0.0116
		(0.0167)		(0.00957)
Avg. Buy-up Coverage	0.189	0.0371**	0.479	0.0110
		(0.0169)		(0.00960)
Buy-up Share	0.186	0.0382**	0.487	0.0116
		(0.0169)		(0.00977)
Total Insured Acreage	0.192	0.0378**	0.491	0.0116
		(0.0169)		(0.00954)
Avg. Month of Loss	0.188	0.0391**	0.488	0.0118
		(0.0167)		(0.00954)
VPD	0.190	0.0367**	0.491	0.0109
		(0.0159)		(0.0118)

Note: Standard errors, in parentheses, are clustered by year (*** p<0.01, ** p<0.05, * p<0.1, where the p-values are from a two-sided t-test).

Supplementary Table 5: Robustness Checks - Average LCR Warming Impacts for Alternative Empirical Models

Crop	(1) Baseline	(2) MA5	(3) MA10	(4) LongDiff	(5) CrossSection
Corn	0.0381**	0.0278**	0.0207**	0.0372	0.0233***
	(0.0167)	(0.0121)	(0.00956)	(0.0326)	(0.00307)
Soybeans	0.0115	0.0205***	0.0116*	0.0393*	0.0331***
	(0.00962)	(0.00700)	(0.00650)	(0.0223)	(0.00556)
Observations					
Corn	30,261	21,288	13,649	3,240	1,733
Soybeans	29,014	20,963	13,771	3,074	1,632

Note: Standard errors in parentheses. For (1)-(3), standard errors are clustered by year, and for (4) and (5), standard errors are clustered by state (*** p<0.01, ** p<0.05, * p<0.1, where the p-values are from a two-sided t-test).

Supplementary Table 6: Simulation analyses on using the county-level yield to predict LCR

	Model used	No. of farms	No. of years	Heteroskedasticity	True LCR	Predicted LCR
(1)	County-level yield	1	100	No	0.124	0.111
(2)	County-level yield	1	30	No	0.124	0.115
(3)	County-level yield	1	15	No	0.124	0.11
(4)	County-level yield	1	100	Yes	0.134	0.106
(5)	County-level yield	1	30	Yes	0.134	0.106
(6)	County-level yield	1	15	Yes	0.134	0.105
(7)	County-level yield	5	100	Yes	0.134	0.05
(8)	County-level yield	5	30	Yes	0.134	0.048
(9)	County-level yield	5	15	Yes	0.134	0.047
	County-level LCR	5	15	Yes	0.134	0.134

Note: The model "County-level yield" estimates $\bar{y}_t = \alpha + \beta temp_t + \varepsilon_t$ and uses the predicted $\bar{y}_t$ to compute the predicted LCR, where $\bar{y}_t$ is the county-level average yield. The model "County-level LCR" estimates $L\bar{C}R_t = \alpha + \beta temp_t + \varepsilon_t$ and directly predicts the LCR.

Supplementary Discussion

Federal Crop Insurance Background

In the United States (US), government subsidized crop insurance has become increasingly important to crop producers (Miranda and Farrin 2012; Coble and Barnett 2013; Goodwin 2015). With substantial growth in subsidy rates, participation rates in crop insurance program increased rapidly (Glauber 2013). In 2014, more than 87% of corn and soybean acres were covered by crop insurance, with liabilities totaling in excess of $70 billion, a more than three-fold increase since 2000 (RMA 2016). The current political landscape surrounding the 2018 Farm Bill suggests that crop insurance will continue to serve as the cornerstone of public support for agricultural production (Barnaby and Russell 2016).

Federal crop insurance in the US was first developed in the 1930s as a response to the crop losses incurred in the Dust Bowl. Over the years, it has changed significantly, with major expansions and modifications in 1980, 1994, 2000, and 2008. The Federal Crop Insurance Corporation (FCIC), which was founded to carry out the insurance program, is run by the Risk Management Agency (RMA). The RMA develops insurance products and sets the premium rates. Private companies can also develop insurance products with the approval of FCIC. In setting the premium rates, the RMA targets actuarially fair levels; that is, levels at which expected total premiums are equal to expected total indemnities. Over the years, the rate-setting procedure has been modified to better accomplish this goal (Goodwin 1994; Glauber 2013).

The individual policies themselves are sold and administered by sixteen approved private insurance companies. There are essentially two types of policies: (i) yield-based (APH) insurance and (ii) revenue-based insurance. Yield-based and revenue-based insurance are further separated into individual-based and group or area-based insurance. Yield insurance protects against the event that yields fall below expected yield, while revenue insurance protects against a combination of yield and prices. Indemnities are paid when yields or revenues fall below the coverage level. For yield insurance, the threshold is with respect to historical yields, whereas with revenue insurance the threshold is based both on historical yields and a projected output price. It is therefore possible in the case of revenue insurance that even in years when yields are quite high that indemnities are paid if the harvest price is low relative to the projected price.

Robustness checks

This section presents additional analyses based on several alternative modeling procedures. We first analyze the robustness of our warming impacts with respect to our temperature variables. We consider three alternatives: (i) including cold temperature variables such as time exposure below 0 °C and degree days between 0 and 10 °C, (ii) expanding the growing season from March to October, and (iii) expanding the growing season from March to October while also including the cold temperature variables. As demonstrated in Supplementary Table 2, the warming impacts are robust to these changes, and we observe greater responsiveness in the cold-related LCR when we expand the growing season and include the cold temperature variables.

We also check whether our main results are driven by a particular insurance product. We estimate the LCR warming effects separately for yield policies and revenue policies (see Glauber (2013) for the description on how each type of policy works). Supplementary Table 3 provides the estimated warming effects from the different two samples: a) yield policies only and b) revenue policies only. While we observe moderately higher impacts in the results for revenue policies only, in general, the results are qualitatively consistent across the different types of insurance products.

We also consider whether our main findings are confounded by not including variables that describe changes in crop insurance participation, as well as not including other weather-related variables such as Vapor Pressure Deficit (VPD). The specific variables we add include: (i) the county average coverage level of purchased policies, (ii) the ratio of “buy up” to “catastrophic loss” policies, (iii) total acreage enrolled, (iv) the loss-weighted average month of the losses, and (v) VPD. Using these variables, we es-

timate five different models by adding each additional control variable to our preferred specification. We then generate out-of-sample predictions by bootstrapping our sample 1,000 times. In each repetition, we use 80% of the bootstrapped sample as the training dataset and the remaining 20% as the test dataset. We report the out-of-sample root mean squared errors (RMSE) and the warming impacts in table 4. We find that there is little difference in the RMSEs across the six models, including our preferred specification, and the warming impacts are highly robust.

Finally, we also provide robustness checks related to the possibility of adaptation. A common criticism of yield impact studies that use panel based empirical models is that the estimates do not account for farmer adaptation to climate (Burke et al. 2016; Hsiang 2016). This same critique can be applied to our approach and is perhaps even more relevant given that the liabilities change on a continual basis in response to changes in yield histories and changes in the FCIP. To assess the importance of adaptation and possible changes in insured liabilities, we follow the approach taken in Hsiang (2016). Specifically, we estimate several different empirical models that filter the data over different intervals and then simulate a uniform 1 °C warming scenario. At one end of the spectrum is the panel method—i.e., the model specified in equation (1)—in which the effects of weather variables are identified by time series variation within each county. At the other end of the spectrum is the cross section model, which purely relies on variation across counties (e.g., as in Mendelsohn et al. 1994). The advantage of the cross section model is that it can capture adaptation to long-run expectations, but this comes at the cost of exposing the estimates to the omitted variable bias caused by not controlling for time-invariant county-specific characteristics. In between the panel and cross section models are the long difference approach and models in which the variables are filtered over multi-year periods. The advantage of these models is that they allow for adaptation (at the level of filtering) while controlling for omitted variable bias, but in filtering the data there is loss of information.

The warming impacts for each model are presented in Supplementary Table 5; for comparison, column (1) provides our baseline warming impacts (the panel approach). The specifications in columns (2)-(5) were designed as follows. In column (2) we compute five year moving averages (MA5) of the LCR, temperature, and precipitation variables, estimate equation (1), and then use the estimated coefficients to compute warming impacts. Column (3) is similar but with ten year (MA10) year moving averages instead. Column (4) contains warming impacts for a long-difference specification (LongDiff); we collapsed the sample into two thirteen year periods—1989-2001 and 2002-2014—and then estimated equation ((1)) without the state-specific trends. Finally, column (5) provides impacts based on a cross section specification in which the variables are averaged over the entire 1989-2014 period. Across all specifications, the estimated impacts are of the same sign, but vary in magnitude. For corn, the impacts range from +17% (MA10) to as high as +32% (BaseLine), and for soybeans, the impacts range from +11% (MA10) to +37% (LongDiff). Notably, among all specifications, the baseline soybean impacts are actually the smallest. Overall, while this exercise is not definitive, we interpret these findings as further corroboration that yield risk and premium rates tend to increase in response to warmer temperatures.

Using Yield Data to Quantify the Impact of Warming on Yield Risk

We conduct a simulation to demonstrate that county-level yields, defined as average yields across farms within a county, cannot capture changes in yield risk from warming temperatures. Farm-level yields are simulated according to:

(1) $$y_{it} = \alpha + \beta temp_t + \varepsilon_{it}.$$

We assume that all $i = 1, ..., I$ farms within a county have the same mean yield and are exposed to the same temperature outcome ($temp_t$), which are drawn from a Uniform(0,10) distribution. Farms differ by their idiosyncratic shock (ε_{it}), which are independently drawn from a Normal(0,σ^2) distribution. The parameters are calibrated such that each farm has a mean yield of 150 bu/acre and a standard deviation of 60, the latter of which was chosen to induce a loss cost ratio (LCR) of 0.10 at a coverage level of 80%. The coefficient on temp (β) was fixed at -10 so that a one unit change in temperature generates a 10 unit

change in yield. This reflects a 6.6% yield loss from warming temperatures which is consistent with the previous literature.

The true LCRs are fixed by assuming a single farm, drawing yields one million times, and averaging over LCRs from each draw, where each LCR is defined as

$$LCR_t = \frac{\{\max y_t^g - y_t, 0\}}{y_t^g}. \tag{2}$$

where y_t^g is the yield guarantee based on a five year average of yield outcomes. To simulate yields under a warming climate, we simply increase the temperature outcome by 1 unit within each draw. The associated LCRs for the baseline and warming climate are 0.110 and 0.124, respectively.

As noted, our goal is to use these yield simulations to evaluate whether county-level yields are able to capture changes in risk, or sensitivity, from a warming climate. First, we define the county-yield as $y_t^c = (1/I)\sum_i y_{it}$ and run a regression of y_t^c on $temp_t$ and an intercept for each of the simulations. The estimated parameters are then used to predict the yield outcomes, denoted by $\hat{y}_t^c$, by replacing $temp_t$ with $(temp_t + 1)$ in each year. The predicted LCR in each year is therefore given by:

$$LCR_t = \frac{\max\{\hat{y}_t^g - \hat{y}_t^c, 0\}}{\hat{y}_t^g}. \tag{3}$$

where $\hat{y}_t^g$ is the yield guarantee based on a five year average of yield outcomes under the warming scenario. The first three rows of Supplementary Table 6 reports the true LCR for the warming climate alongside the predicted LCR based on the county-level yield (CLY) regression model, averaged across 100 simulations, where each sample is constructed assuming yield histories of T = 15, 30, and 100 years. For this particular case, where there is a single farm within the county, the CLY model accurately predicts the LCR for small and large sample sizes.

Next we consider the more realistic scenario where temperature affects both the mean and variance of yields. The simulated data is constructed as before, except that now the variance of the error term depends on the temperature outcome according to: $\sigma^2 = \delta temp_t$. The value of δ is fixed at 64, which induces a baseline LCR of 0.103 and warming LCR of 0.134, both consistent with the values estimated in this paper. The CLY model is not updated to account for this temperature-induced heteroskedasticity, and we find that its LCR predictions understate the true effect warming on yield risk (rows (4) – (6), Supplementary Table 6).

So far the simulations have assumed a single farm, effectively equalizing farm and county-level yields. We next consider the effects of aggregating across $I = 5$ farms within the county and find that the CLY model under-predicts the warming LCR substantially, demonstrating the inability of county-level averages to predict warming effects on risk (rows (7) – (9), Supplementary Table 6). Finally, we include the model used in the paper based on county-level LCRs (CLLCR) and find that it accurately predicts the warming LCR. In sum, we find that estimating the conditional mean equations using county-level average yields leads to poor predictions of the loss-cost ratio, thus leading to incorrect inferences about the impacts of temperature on yield risk.

Supplementary References

Barnaby, G. A. and L. Russell (2016). Theme overview: Crop insurance in the 2018/2019 farm bill. *Choices 31*(3).

Burke, M., K. Emerick, et al. (2016). Adaptation to climate change: Evidence from us agriculture. *American Economic Journal: Economic Policy 8*(3), 106–40.

Coble, K. H. and B. J. Barnett (2013). Why do we subsidize crop insurance? *American Journal of Agricultural Economics 95*(2), 498–504.

Glauber, J. W. (2013). The growth of the federal crop insurance program, 1990–2011. *American Journal of Agricultural Economics 95*(2), 482–488.

Goodwin, B. K. (1994). Premium rate determination in the federal crop insurance program: What do averages have to say about risk? *Journal of Agricultural and Resource Economics*, 382–395.

Goodwin, B. K. (2015). Agricultural policy analysis: the good, the bad, and the ugly. *American Journal of Agricultural Economics 97*(2), 353–373.

Hsiang, S. (2016). Climate econometrics. *Annual Review of Resource Economics 8*, 43–75.

Mendelsohn, R., W. D. Nordhaus, and D. Shaw (1994). The impact of global warming on agriculture: a ricardian analysis. *The American Economic Review*, 753–771.

Miranda, M. J. and K. Farrin (2012). Index insurance for developing countries. *Applied Economic Perspectives & Policy 34*(3).

RMA (2016). Summary of business. `http://www.rma.usda.gov/data/sob.html`.
