# Supplementary material for: Using insurance data to quantify the multidimensional impacts of warming temperatures on yield risk
Source: Nat Commun. 2020 Sep 11;11:4542. doi: 10.1038/s41467-020-17707-2 (PMC7486405; doi:10.1038/s41467-020-17707-2)
Supplement: Supplementary file 3 — Reporting Summary [file 41467_2020_17707_MOESM3_ESM.pdf]

nature research

Corresponding author(s): Edward Perry

Last updated by author(s): 05/29/2020

Reporting Summary

Nature Research wishes to improve the reproducibility of the work that we publish. This form provides structure for consistency and transparency in reporting. For further information on Nature Research policies, see our Editorial Policies and the Editorial Policy Checklist.

Statistics

For all statistical analyses, confirm that the following items are present in the figure legend, table legend, main text, or Methods section.

n/a	Confirmed	
☐	☒	The exact sample size (*n*) for each experimental group/condition, given as a discrete number and unit of measurement
☒	☐	A statement on whether measurements were taken from distinct samples or whether the same sample was measured repeatedly
☐	☒	The statistical test(s) used AND whether they are one- or two-sided<br>*Only common tests should be described solely by name; describe more complex techniques in the Methods section.*
☐	☒	A description of all covariates tested
☐	☒	A description of any assumptions or corrections, such as tests of normality and adjustment for multiple comparisons
☐	☒	A full description of the statistical parameters including central tendency (e.g. means) or other basic estimates (e.g. regression coefficient) AND variation (e.g. standard deviation) or associated estimates of uncertainty (e.g. confidence intervals)
☐	☒	For null hypothesis testing, the test statistic (e.g. *F*, *t*, *r*) with confidence intervals, effect sizes, degrees of freedom and *P* value noted<br>*Give P values as exact values whenever suitable.*
☒	☐	For Bayesian analysis, information on the choice of priors and Markov chain Monte Carlo settings
☒	☐	For hierarchical and complex designs, identification of the appropriate level for tests and full reporting of outcomes
☒	☐	Estimates of effect sizes (e.g. Cohen's *d*, Pearson's *r*), indicating how they were calculated

Our web collection on statistics for biologists contains articles on many of the points above.

Software and code

Policy information about availability of computer code

Data collection	All data were collected from publicly available websites. The Cause of Loss and Summary of Business datasets can be obtained at https://www.rma.usda.gov/SummaryOfBusiness/CauseOfLoss. The PRISM weather data can be obtained at http://www.columbia.edu/~ws2162/links.html.
Data analysis	All statistical and regression analyses were conducted using the statistical software StataSE 15.

For manuscripts utilizing custom algorithms or software that are central to the research but not yet described in published literature, software must be made available to editors and reviewers. We strongly encourage code deposition in a community repository (e.g. GitHub). See the Nature Research guidelines for submitting code & software for further information.

Data

Policy information about availability of data

All manuscripts must include a data availability statement. This statement should provide the following information, where applicable:

- Accession codes, unique identifiers, or web links for publicly available datasets
- A list of figures that have associated raw data
- A description of any restrictions on data availability

The Cause of Loss and Summary of Business datasets can be obtained at https://www.rma.usda.gov/SummaryOfBusiness/CauseOfLoss. The PRISM weather data can be obtained at http://www.columbia.edu/~ws2162/links.html and at \url{http://prism.oregonstate.edu. Further details concerning preparation of the data are available at https://github.com/jisangyu-agecon/LCR_Perry_et_al.git.

Field-specific reporting

Please select the one below that is the best fit for your research. If you are not sure, read the appropriate sections before making your selection.

☐ Life sciences ☐ Behavioural & social sciences ☒ Ecological, evolutionary & environmental sciences

For a reference copy of the document with all sections, see nature.com/documents/nr-reporting-summary-flat.pdf

Ecological, evolutionary & environmental sciences study design

All studies must disclose on these points even when the disclosure is negative.

Study description	We use publicly available weather and crop insurance data to assess the impact of warming on the magnitude of yield risk and its causes.
Research sample	The crop insurance data were obtained from the Summary of Business (SOB) and Cause of Loss (COL) databases, both publicly available datasets maintained by the RMA. The SOB data contains county-year specific observations of total losses and liabilities by crop. The COL data contains crop-county-year specific observations of losses disaggregated by each of 44 different possible causes. We restrict our analysis to corn and soybeans from 1989-2014. These are the two most widely grown and insured crops in the United States, and 1989-2014 is the period for which we could obtain the required information on crop insurance. Precipitation and weather data come from the Parameter-elevation Regressions on Independent Slopes Model (PRISM) dataset. We compute daily degree days above each 1 °C threshold using the sinusoidal curve method 59 and then aggregate degree days that fall within the assumed growing season of April to September.
Sampling strategy	We use the annual county-level aggregated data, which are based on the population of all insured corn and soybeans farms. Given that we use publicly available aggregated data, no sample size calculation is required.
Data collection	We use publicly available data from the two sources we describe in the manuscript: USDA, RMA, and NOAA. EP and JY downloaded and proceed data. The codes are either available at the provided github link or available upon request.
Timing and spatial scale	U.S. dryland counties (east of 100th meridian) that produce corn or soybeans from 1989 to 2014. We are utilizing all available time periods and we focus on dryland counties following the literature represented by Schlenker and Roberts (2009).
Data exclusions	No excluded data within the spatial and temporal coverage described above.
Reproducibility	We provide the reproducing codes (Stata do files) and we also provide a link for the cleaned dataset.
Randomization	N/A
Blinding	N/A

Did the study involve field work? ☐ Yes ☒ No

Reporting for specific materials, systems and methods

We require information from authors about some types of materials, experimental systems and methods used in many studies. Here, indicate whether each material, system or method listed is relevant to your study. If you are not sure if a list item applies to your research, read the appropriate section before selecting a response.

Materials & experimental systems

n/a	Involved in the study
☒	☐ Antibodies
☒	☐ Eukaryotic cell lines
☒	☐ Palaeontology and archaeology
☒	☐ Animals and other organisms
☒	☐ Human research participants
☒	☐ Clinical data
☒	☐ Dual use research of concern

Methods

n/a	Involved in the study
☒	☐ ChIP-seq
☒	☐ Flow cytometry
☒	☐ MRI-based neuroimaging
